# Skin cancer risk factors among Black South Africans—The Johannesburg Cancer Study, 1995–2016

**DOI:** 10.1002/iid3.623

**Published:** 2022-06-06

**Authors:** Babongile C. Ndlovu, Mazvita Sengayi‐Muchengeti, Caradee Y. Wright, Wenlong C. Chen, Lazarus Kuonza, Elvira Singh

**Affiliations:** ^1^ South African Field Epidemiology Training Program National Institute for Communicable Diseases, Division of the National Health Laboratory Service Johannesburg South Africa; ^2^ National Cancer Registry, National Health Laboratory Service Johannesburg South Africa; ^3^ School of Public Health, Faculty of Health Sciences University of the Witwatersrand Johannesburg South Africa; ^4^ South African DSI‐NRF Centre of Excellence in Epidemiological Modeling and Analysis (SACEMA) Stellenbosch University Stellenbosch South Africa; ^5^ Environment and Health Research Unit South African Medical Research Council Pretoria South Africa; ^6^ Department of Geography, Geoinformatics and Meteorology University of Pretoria Pretoria South Africa; ^7^ Sydney Brenner Institute for Molecular Bioscience, Faculty of Health Sciences University of the Witwatersrand Johannesburg South Africa

**Keywords:** Black population, keratinocyte skin cancer, melanoma skin cancer, risk factors, South Africa

## Abstract

**Background:**

The Black population has lower skin cancer incidence compared to White, Indian/Asian, and Mixed‐race populations in South Africa; however, skin cancer still exists in the Black population. The aim of this study is to identify risk factors associated with skin cancer among Black South Africans.

**Materials and Methods:**

A case‐control study was conducted. Cases were patients with keratinocyte cancers (KCs) and/or melanoma skin cancers (MSCs) and controls were cardiovascular patients. Sociodemographic exposures, environmental health variables, smoking, and HIV status were assessed. Stepwise logistic regression was used to identify risk factors associated with KCs and MSCs.

**Results:**

The KCs histological subtypes showed that there were more squamous cell carcinomas (SCCs) (78/160 in females, and 72/160 in males) than basal cell carcinomas (BCCs). The SCC lesions were mostly found on the skin of the head and neck in males (51%, 38/72) and on the trunk in females (46%, 36/78). MSC was shown to affect the skin of the lower limbs in both males (68%, 27/40) and females (59%, 36/61). Using females as a reference group, when age, current place of residency, type of cooking fuel used, smoking, and HIV status were adjusted for, males had an odds ratio (OR) of 2.04 for developing KCs (confidence interval [CI]: 1.08–3.84, *p* = .028). Similarly, when age, current place of residency, and place of cooking (indoors or outdoors) were adjusted for, males had an OR of 2.26 for developing MSC (CI: 1.19–4.29, *p* = .012).

**Conclusions:**

Differences in the anatomical distribution of KCs by sex suggest different risk factors between sexes. There is a positive association between being male, smoking, rural dwelling, and a positive HIV status with KCs and being male and rural dwelling with MSC. The rural dwelling was a newly found association with skin cancer and warrants further investigation.

## Graphical abstract

Differences in the anatomical distribution of skin cancer by sex suggest different risk factors between sexes. The rural dwelling was a newly found association with skin cancer and warrants further investigation.

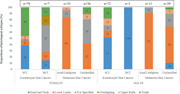

## 1 INTRODUCTION

In South Africa (SA), White, Mixed‐race, and Indian/Asian population groups have higher skin cancer incidence compared to the Black population.^1^, ^2^ A study by Norval et al.^3^ on the incidence of skin cancers in the population groups of SA from 2000 to 2004 showed that the White population is the most susceptible population group to skin cancer, followed by the Mixed‐race population, Indian/Asian population, then the Black population. The age‐standardized incidence rates of keratinocyte cancers (KCs) were reported to be 1.6–3.0 per 100,000 in the Black, population and 8.5–51.3 per 100,000 in the White population in SA.^3^ Common misperceptions about skin cancer exist among the Black population including the belief that having darker skin (i.e., abundant melanin skin pigmentation) offers full protection against skin cancer; however, it has been shown that the Black population is also susceptible to skin cancer.^4^, ^5^ The incidence rates of skin cancer are relatively well documented in SA but the risk factors associated with skin cancer, especially in the Black population have not been explored.^3^

The high burden of HIV infection in the Black population of SA leads to susceptibilities to many diseases (including cancer) due to immunosuppression.^6^, ^7^, ^8^, ^9^ In the antiretroviral era, HIV‐positive patients live longer and are thus susceptible to other diseases like cancer.^6^, ^7^, ^8^, ^9^ Squamous cell carcinoma (SCC) subtype of KCs has been shown to be associated with HIV infection (odds ratio [OR]: 2.6, 95% confidence interval, CI [1.4–4.9]).^10^ Skin cancer is therefore of public health concern in SA due to a dual risk of the country's high burden of HIV and high ambient UVR environment (the main risk factor for skin cancer).^2^ Understanding skin cancer incidence patterns and etiology in the South African Black population, the largest population group in SA, is critical for planning, prevention, treatment strategies, and allocation of medical resources.^2^ The aim of this study is to describe the histological subtypes and anatomical distribution of skin cancer subtypes and identify risk factors associated with skin cancer among Black South Africans.

## 2 MATERIALS AND METHODS

Using data from the Johannesburg Cancer Study (JCS), we conducted a case‐control study where cases were Black patients with a confirmed diagnosis of an invasive skin cancer (KCs or melanoma skin cancers [MSCs]). There were 160 KCs and 101 MSCs from the JCS. The participants self‐reported as black, and the ability to speak one of the main languages in SA was the inclusion criteria. Self‐identified black but nonresident South African patients were excluded. The JCS was established in 1995 at the National Cancer Registry of SA. Its original aims were to examine whether risk factors identified for cancer in Western countries applied to black patients in Johannesburg, SA, and to understand the impact of HIV on cancer risk, with a view to identify previously unrecognized HIV‐associated cancers.^11^ The JCS recruited adult (18+ years old) consenting, self‐identified Black patients, who were newly diagnosed with cancer and attending public referral hospitals for oncology and radiation therapy in Johannesburg Tertiary Hospitals (Chris Hani Baragwanath Academic Hospital and Charlotte Maxeke Johannesburg Academic Hospital, and its affiliated Radiation Oncology ward of Hillbrow Hospital).^11^ The JCS collected sociodemographic data and environmental exposure data by conducting face‐to‐face interviews, using a paper‐based questionnaire which was administered by qualified nurses. Data were collected from 1995 to 2016.^11^ Between 1998 and 2001 JCS recruited patients presenting with cardiovascular diseases as a cancer‐negative control group. Anonymized data were obtained and each participant was assigned a unique study number.

The exposure variables of interest that were analyzed in this study were sociodemographic variables (i.e., sex, age, rural/urban (termed urbanicity) dweller in the province of birth, rural/urban dweller at the current province of residency, and level of education), environmental health variables (i.e., type of walls of the house the patient lives in, whether they cook indoors/outdoors currently and in the past, type of cooking fuel used currently and in the past, and type of fuel used for heating currently and in the past), lifestyle variables, that is, smoking status (current smoker–smoking within current 5 years, past smoker–smoked in the past 5–10 years, or nonsmoker–never smoked), snuff use, and HIV status (tested at the time of the study).

Proportions of cases were described according to the two major skin cancer groups (KCs and MSCs) and stratified by sex. The analysis defined proportions between males and females by the number of cases, median age (interquartile range [IQR]), and 10‐year category age grouping. The skin cancers were further described by histological subtypes and anatomical sites (i.e., the skin of: “head and neck,” “lower limbs,” “overlapping” (could be in multiple anatomical categories), “upper limbs” and “trunk”). The “not disclosed” (i.e., “not specified” or “unknown”)–refers to unassigned skin regions (the medical records not specifying which part of the skin has the lesion). We performed a stepwise (backward elimination) regression analysis to identify factors associated with each skin cancer subtype. A multivariable, adjusted model was presented, separately for KCs and MSCs. Analyses were performed using Stata version 15 (Stata Corp Ltd).

Approval to conduct the study was obtained from the Human Research Ethics Committee (Medical) of the University of the Witwatersrand, clearance certificate number: M181191. Permission was obtained from the respective proprietors of the primary dataset.

## 3 RESULTS

### 3.1 Demographics

There were 160 KCs cases and 53% (*n* = 85) were females. The males with KCs were older than females: 51 (IQR: 41–59) versus 46 (IQR: 36–56) years. Most cases were recorded in the age group 51–60 years (28%, 44/160). There were 101 MSC cases and 60% (*n* = 61) were females. The median age at diagnosis was similar in both males: 55 (IQR: 49–68) and females: 56 (IQR: 47–68) years. Most MSC cases were recorded in the age group 51–60 years (28%, 28/101), see Table 1.

**TABLE 1:** Case distribution of keratinocyte cancers and melanoma skin cancer cases in the Johannesburg Cancer Study, 1995–2016

	Keratinocyte cancers			Melanoma skin cancers		
	All	Males	Females	All	Males	Females
*n* (row %)	160 (100)	75 (47)	85 (53)	101 (100)	40 (40)	61 (60)
Age median in years (IQR)	49 (38–57)	51 (41–59)	46 (36–56)	56 (48–68)	55 (49–68)	56 (47–68)
Age groups in years						
18–30	17 (11)	4 (5)	13 (15)	2 (2)	1 (3)	1 (2)
31–40	33 (21)	14 (19)	19 (22)	10 (10)	3 (8)	7 (13)
41–50	40 (25)	19 (25)	21 (25)	21 (21)	9 (3)	12 (20)
51–60	44 (28)	26 (35)	18 (21)	28 (28)	12 (30)	16 (27)
61–70	21 (13)	10 (13)	11 (13)	17 (17)	7 (18)	10 (16)
71–80	3 (2)	1 (1)	2 (2)	17 (17)	5 (13)	12 (20)
81+	2 (1)	1 (1)	1 (1)	6 (6)	3 (8)	3 (5)
HIV status						
Positive	48 (30)	16 (21)	32 (38)	18 (18)	8 (20)	10 (16)
Negative	83 (52)	48 (64)	35 (41)	71 (70)	29 (72)	42 (69)
No response	29 (18)	11 (15)	18 (21)	12 (12)	3 (8)	9 (15)
Smoking						
Currently smoking	52 (33)	44 (59)	8 (9)	17 (17)	15 (38)	2 (3)
Smoked in the past (5–10 years)	36 (23)	19 (25)	17 (20)	13 (13)	7 (18)	6 (10)
Never smoked	71 (44)	12 (16)	59 (69)	70 (69)	18 (45)	52 (85)
No information on smoking	1 (0.6)	0 (0)	1 (1)	1 (1)	0 (0)	1 (2)

*Note*: Numbers may not add to 100% due to rounding.

Abbreviation: IQR, interquartile range.

### 3.2 Histological subtypes and anatomical site

**FIGURE 1:**
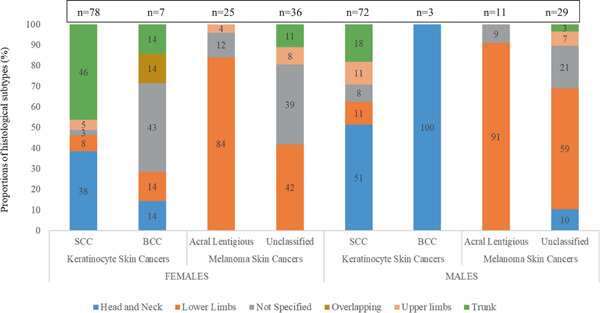
Histological subtypes and anatomical sites of distribution of skin cancers in the Johannesburg Cancer Study, 1995–2016. Sites include “head and neck,” “lower limbs,” “not specified” (i.e., unknown), “overlapping” (could be in multiple anatomical categories), “upper limbs,” and “trunk.” BCC, basal cell carcinoma; SCC, squamous cell carcinoma.

There were more SCCs in both females (*n* = 78) and males (*n* = 72) compared to basal cell carcinoma (BCC) (female: 7; male: 3). The distribution of SCCs in females showed that most cases were recorded on the skin of the trunk, 36/78 (46%) and skin of the head and neck, 30/78 (38%). In males 37/72 (51%), SCCs were recorded on the skin of the head and neck and 13/72 (18%) on the skin of the trunk. MSC was recorded as the most common on the skin of lower limbs in both females and males, 36/61 (59%) and 27/40 (68%), respectively, see Figure 1. Thirty‐six percent (36/101) of melanomas were classified as acral lentiginous melanoma in both males and females combined, and the rest of the melanomas were not classified. The distribution of acral lentiginous melanomas showed that most lesions were recorded from the skin of lower limbs in both females (84%, 21/25) and males (91%, 10/11), see Figure 1.

### 3.3 Risk factor analysis: KCs

In the univariable analysis, males were shown to have an OR of 2.56 for developing KCs when compared to females (CI: 1.60–4.10, *p* < .001) (Table 2). When age, current urbanicity, type of cooking fuel used currently, smoking, and HIV status were adjusted for, males had an OR of 2.04 for having KCs (CI: 1.08–3.84, *p* = .028) (Table 2). This model explained 11% of the variation observed in KCs outcome. Significant associations of smoking and HIV infection with KCs were found: current smokers (OR = 2.65, CI: 1.24–5.64, *p *= .012) and HIV‐positive (OR = 2.00, CI: 1.09–3.53, *p *= .024). Marginal significance of urbanicity with KCs was observed in rural dwellers (OR = 4.5, CI: 1.58–13.06, *p *= .005). Age was kept in the final model for face validity, as it is known as a risk factor for cancer and should always be adjusted for in models.

**TABLE 2:** Univariable and multivariable logistic regression of keratinocyte skin cancer risk factors in black South Africans, Johannesburg Cancer Study 1995–2014

	Univariable logistic regression (*p* ≤ .25)			Multivariable logistic regression analysis (*p* ≤ .05)		
	Unadjusted odds ratios	95% Confidence intervals	*p*	Adjusted odds ratios	95% Confidence intervals	*p*
Sociodemographic variables	*n* = 320, *R* ^2^ = 0.0356, *p* = .000			*n* = 293, *R* ^2^ = 0.1133, *p* = .000		
Gender (*n* = 320)						
Female (ref)	1			1		
Male	2.56	1.60–4.10	.000	2.04	1.08–3.84	**.028**
Age (*n* = 320)	0.98	0.97–1.00	.040	0.99	0.97–1.01	.591
Rural/urban dweller currently (*n* = 316)						
Urban (ref)	1			1		
Rural	2.70	1.25–5.84	.011	4.5	1.58–13.06	**.005**
Environmental variables						
Place of cooking currently (*n* = 313)						
Inside (ref)	1			1		
Outside	5.10	0.59–44.15	.139	7.42	0.50–110.90	.146
Type of cooking fuel used currently (*n* = 316)						
Electricity (ref)	1			1		
Wood	1.46	0.45–4.73	.528	0.16	0.02–1.13	.065
Coal	0.91	0.43–1.95	.814	0.69	0.28–1.71	.422
Paraffin/gas	1.53	0.76–3.09	.236	0.92	0.41–2.09	.845
Behavioral variables						
Smoking (*n* = 316)						
Never smoked (ref)	1			1		
Smoked in the past (5–10 years)	1.60	0.91–2.81	.103	1.73	0.88–3.39	.113
Current smoker	3.08	1.74–5.46	.000	2.65	1.24–5.64	**.012**
HIV status (*n* = 300)						
Negative (ref)	1					
Positive	1.72	1.06–2.78	.028	2.00	1.09–3.53	**.024**

*Note*: Bold values are statistically significant at *p* < .05.

### 3.4 Risk factor analysis: MSC

In the univariable analysis, males were shown to have OR of 1.99 for having MSC when compared to females (CI: 1.09–3.64, *p* = .025) (Table 3). When age, current urbanicity, and place of cooking (indoors or outdoors) were adjusted for, males had an OR of 2.26 for having MSC (CI: 1.19–4.29, *p* = .012) (Table 3). This model explained 9% of the variation observed in MSC outcomes (Table 3). A significant association between living in rural areas was found with a higher risk among rural dwellers (OR = 2.88, CI: 1.01–8.18, *p *= .048) versus urban dwellers. The place of cooking was kept in the model because it served as a proxy for sun exposure.

**TABLE 3:** Univariable and multivariable logistic regression of melanoma skin cancer risk factors in black South Africans, Johannesburg Cancer Study 1995–2014

	Univariable logistic regression (*p* ≤ .25)			Multivariable logistic regression analysis (*p* ≤ .05)		
	Unadjusted odds ratios	95% Confidence intervals	*p*	Adjusted odds ratios	95% Confidence intervals	*p*
Sociodemographic variables	*n* = 202, *R* ^2^ = 0.0183, *p* = .023			*n* = 201, *R* ^2^ = 0.0890, *p* = .000		
Gender (*n* = 165)						
Female (ref)	1			1		
Male	1.99	1.09–3.64	.025	2.26	1.19–4.29	**.012**
Age (*n* = 202)	1.03	1.01–1.06	.001	1.04	1.02–1.06	**.001**
Rural/urban dweller at current residency (*n* = 201)						
Urban (ref)	1			1		
Rural	3.08	1.23–7.69	.016	2.88	1.01–8.18	**.048**
Environmental variables						
Place of cooking currently (*n* = 201)						
Inside (ref)	1			1		
Outside	3.03	0.31–29.64	.341	1.42	0.12–17.41	1.784

*Note*: Bold values are statistically significant at *p* < .05.

## 4 DISCUSSION

More KCs were observed than MSCs in this study. The majority of KCs lesions were on the skin of the head and neck in males and the skin of the trunk in females. The majority of MSCs were on the skin of the lower limbs with acral lentiginous melanoma being the most observed histological subtype. Being male, HIV infected, living in a rural area, and smoking was positively associated with KCs. Being male and living in a rural area was also positively associated with MSCs. The skin cancer subtype that was reported more frequently in this study population was SCC compared to MSC, consistent with literature that the Black population is mostly affected by SCC.^12^, ^13^, ^14^ However, the distribution of histological subtypes of KCs in the SA general population (all population groups) shows that BCC is the most abundant subtype, followed by SCC.^15^, ^16^ This pattern is due to the overwhelming numbers of BCC diagnosed in the White population in SA. However, when population group‐specific analysis was conducted, SCC was the leading skin cancer in the Black population, followed by BCC.^3^

A risk factor for SCC in the Black population is hypothesized to be immunosuppression resulting from HIV infection.^17^ SA has one of the world's largest HIV epidemics and the Black population is the most affected population.^17^ A rise in SCC incidence in the Black population of SA was observed after the beginning of the HIV epidemic.^17^ Shortly after the introduction of antiretroviral treatment, a significant decline in SCC incidence was observed in SA and this is consistent with the theory that relates SCC to immunosuppression resulting from HIV infection.^17^ The risk factor analysis results showed that being male resulted in double the odds of having skin cancer for both KCs and MSC, as supported by other studies in other countries.^18^, ^19^ Sex differences exist in many physiological conditions and can be explained by various theories.^18^

In this study, a positive association of KCs with smoking (current smoking) was seen.^19^, ^20^, ^21^ Current smokers and persons with a history of smoking have increased odds of being diagnosed with SCC.^19^ Our findings are consistent with the literature on the association between smoking and KCs. The rural dwelling was positively associated with both KCs and MSC. Given sun exposure as a primary risk factor for skin cancer susceptibility (although inconclusive in deeply pigmented skin) and higher odds of skin cancer in males, the association can be explained by the nature of jobs usually done by men (outdoor jobs) and we can assume lack of sun protection, especially on the uncovered areas of the body.^6^, ^22^ In our results on histological subtypes and anatomical sites of skin cancers, it was shown that more than 50% of SCC lesions are on the skin of the head and neck in males. Men usually cut their hair, making their head region susceptible to sun exposure. The Black SA economy relies on agricultural and mining activities which increase exposure to risk factors such as skin injuries and scar tissue, a precursor of skin cancer development.^17^, ^23^

The strength of the current study is that it addresses the existing gap in knowledge of the skin cancer risk factors in the Black population. Even though the study participants were recruited from one province (limiting the generalizability of findings to the SA Black population), the Johannesburg population is essentially a mixture of all SA provinces as Johannesburg is a central hub of economic activity in SA and people migrate from different provinces (from rural areas mostly) to Johannesburg seeking employment during their adult lives.^24^ Our findings were also consistent with existing literature from other settings on the association between KCs and smoking, and KCs and HIV. The limitations of this study are that the study did not collect all risk factor information relating to skin cancer for example; family history of skin cancer, trauma to a site of skin cancer, exposure to sunlight, skin phototype, information on albinism, and iatrogenic status among other factors were not captured. The original study focused on collecting commonly known cancer risk factors from other study settings. The selection of participants in the current study may have underestimated the ratio of skin cancer cases in general, as the sampled cases were only advanced cases that were referred from lower levels of care for radiation therapy.

## 5 CONCLUSION

The differences in the anatomical distribution of KCs by sex suggest different risk factors between sexes and warrant further investigations. Our analysis associates; being male, smoking, rural dwelling, and a positive HIV status with KCs and being male and rural dwelling with MSCs. Our results are in agreement with literature from other settings, where smoking and HIV have been associated with KCs. The rural dwelling was a newly found association with skin cancer and warrants further investigation. These factors are overlapping but different; we, therefore, recommend targeted screening of skin cancers in HIV‐positive patients and smokers, sensitization of rural dwellers to the potential of skin cancer risk and encouragement for screening (early detection), and education about skin cancer in black communities to debug the myth that deeply pigmented skin is not affected by skin cancer.

## AUTHOR CONTRIBUTIONS

All authors have read and approved the final manuscript. Elvira Singh, Mazvita Sengayi‐Muchengeti, and Babongile C. Ndlovu conceptualized the study. Wenlong C. Chen contributed to the study design. Babongile C. Ndlovu, Mazvita Sengayi‐Muchengeti, and Elvira Singh analyzed the data. Elvira Singh, Mazvita Sengayi‐Muchengeti, and Lazarus Kuonza supervised the project. Babongile C. Ndlovu wrote the first draft. All authors contributed to the revision of the manuscript. Caradee Y. Wright provided expert advice during the drafting of the manuscript.

## CONFLICTS OF INTEREST

The authors declare no conflicts of interest.

## Data Availability

The author(s) confirm that they had full access to all the data in the study and take responsibility for the integrity of the data and the accuracy of the data analysis. The dataset for this publication is not publicly available and can be obtained from the corresponding author on request.
